# Implementing a mental fitness workshop in a Canadian Practical Nursing Program: A qualitative descriptive study

**DOI:** 10.1371/journal.pmen.0000760

**Published:** 2026-10-28

**Authors:** Linda Frost, Jana Borras, Ariel Dysangco, Vimbayi Carine Makwaira

**Affiliations:** 1 School of Nursing, York University, Toronto, Ontario, Canada; 2 School of Nursing, Sheridan College, Brampton, Ontario, Canada; PLOS: Public Library of Science, UNITED KINGDOM OF GREAT BRITAIN AND NORTHERN IRELAND

## Abstract

Nursing students experience high levels of stress that can negatively affect mental health and well-being. This study explored practical nursing students’ perceptions of the acceptability and perceived value of a brief mental fitness workshop integrated into nursing education. Two sessions from a condensed version of the Mastering My Mental Fitness for Nurses program were embedded within a fourth‑semester laboratory course. A qualitative descriptive design was used, drawing on reflexive thematic analysis of semi‑structured interviews (n = 5) and free‑text post‑session feedback forms (n = 49). Participants reported enhanced awareness of stress and burnout, reframed self‑care as a professional responsibility, and valued opportunities for peer connection through small‑group discussions. Students recommended earlier and more frequent integration within the curriculum and greater flexibility in delivery. While the findings reflect short‑term perceptions rather than clinical outcomes, the workshops were perceived as feasible and acceptable, with implications for mental health promotion and burnout prevention among nursing students. Future research should examine impact using validated mental health and well-being measures.

## Introduction

The academic environment that nursing students are immersed in comes with many demands and expectations. Course load, clinical placements, and financial expenses are examples of the pressures facing students each semester, which accumulate throughout their nursing education. These pressures contribute to elevated levels of stress among nursing students and can negatively impact their overall well-being and mental fitness [1]. Nursing students experience significant stress across all years of their program, although the sources and intensity of the stress may change as they progress through their studies. Evidence suggests that senior nursing students often report reduced mental well-being in comparison to students in earlier years, likely reflecting the cumulative impact of sustained academic and clinical demands [2,3]. When these stressors persist and are not effectively managed, they may contribute to academic burnout, which has been associated with poorer mental health outcomes and challenges integration into the professional nursing practice [4]. Burnout is defined in the ICD‑11 as an occupational phenomenon resulting from unmanaged workplace stress, characterized by exhaustion, mental distancing or cynicism, and reduced professional efficacy [5].

Mental health promotion is a broader framework that focuses on empowering individuals to take greater control over their health and well-being by strengthening personal, psychological, and social resources, with the goal of building resilience and supporting an individual’s ability to manage life challenges and stressors [6,7]. Within this framework, mental fitness can be understood as a set of modifiable skills and capacities to support positive psychological functioning. We define mental fitness as the individual’s capacity to draw on personal skills and available resources to facilitate goal-directed behaviour, effective self-regulation, and recovery from stress and challenges [8]. Students’ ability to cope with stress and bounce back from adversity is enhanced when their mental fitness is strengthened. Building mental fitness has the benefit of improving physical health, relationships, self-esteem, resilience, work productivity, and overall contentment, happiness and life satisfaction [9]. At its core, building mental fitness is about developing strategies, skills, and tools needed to effectively manage stress, maintain well-being, and function in demanding environments. It encompasses the understanding of one’s mental health, being aware of thinking and behaviour patterns, and engaging in strategies to improve mood and outlook. Mental fitness involves an individual’s capacity to deliberately choose how they respond to situations and circumstances and supports their ability to pause, stay calm, and respond in a healthy and positive manner, rather than in anger, frustration, or other negative reactions [10].

Given the current challenging student landscape, an opportunity exists to better prepare nursing students to effectively cope with the demands and stressors that are inherent to the nursing profession. With the prevalence of burnout in nursing students, nursing faculty are encouraged to develop strategies to enhance students’ skills in areas such as self-efficacy to decrease academic burnout [11]. Providing this group of vulnerable students with workshops that include coping strategies can provide benefits to mental wellness and clinical performance [12]. Literature identifies a positive impact for post-secondary students attending mental wellness courses, but largely these courses were provided to non-nursing students, such as medical, social work and psychology students [13,14]. However, the studies that were focused on nursing students, were found to take place in universities outside of Canada [15,16]. The present study addresses this gap by examining the feasibility and perceived value of integrating a brief mental fitness workshop into a practical nursing program in Canada. This qualitative study was conducted to examine the following question: “What are practical nursing students’ perceptions of the acceptability, relevance, and perceived mental health impact of embedded mental fitness workshops?” This study is timely given growing recognition of the mental health challenges experienced by nursing students and will provide insight into how a health initiative integrated into the nursing program might support students’ mental fitness and well-being.

## Methods

### Ethics statement

Study approval was obtained from Sheridan Research Ethics Board (Approval #2023-10-001-026). Written informed consent was obtained from all interview participants and completion of anonymous post-workshop feedback forms constituted implied consent. Participation was voluntary, non-remunerated, and had no impact on course standing or academic evaluation. Participant confidentiality was maintained through de-identification of all data prior to analysis.

### Description of the workshops

The Mastering My Mental Fitness for Nurses (MMMF-N) workshops were created and facilitated by our external partner Betty Franklin., a Registered Nurse, who founded Let It Begin with Me Inc. The researchers did not receive financial benefit from the intervention, and the study was conducted independently of program commercialization. The workshops used in this study represent a condensed version of the original MMMF-N program, which was developed as a 12-week, 39-hour curriculum. The adapted version consisted of two 90-minute in-person workshops. The intention of the workshops was to contribute to the development of skills and strategies that support nursing students in responding to school and life challenges while maintaining their mental fitness. The workshops were embedded into weeks 1 and 10 of the nursing Lab 4 skills day. The workshops focused on interactive health promoting strategies to support student’s mental fitness and well-being and addressed topics such as personal and professional values, self-esteem, healthy boundaries, mental health vulnerabilities and stigma, feelings and emotional regulation (see Table 1). Participants were encouraged to think reflectively, critically, and creatively about how they will support and care for themselves throughout a career that can erode the very reason they became a healthcare professional.

**Table 1:** MMMF-N Workshop Topics.

Workshop Theme	Class Activities
Introduction to the MMMF-N Program	• Why I Chose to Become a Nurse • The Profession of Nursing and Stress • Staying Fit to Practice, a Salutogenic Approach, and PRESSing In (Personal Responsibility, Education, Self-Advocacy, Support) • The Art and Science of Health and Well-being • Being Aware of Who I Am • Being Aware of What I Do - The “F Words” in My Life (using the Six F’s of Health and Well-being framework - Family, Fitness, Friends, Finance, Fun, and Faith) • My Thinking Scoreboard (being aware of my thoughts using the Six “F Words”)
Caring for You and Caring for Others	• Identifying My Stressors (using the framework of the Six “F Words”) • The Mental Health Continuum and the importance of our thoughts (our mental mindset as our greatest asset, neuroplasticity, and moving from surviving to thriving) • Identifying self-care strategies and developing my personal Mastering My Mental Fitness® action / self-care plan (using the Six “F Words”)

### Design and participants

A qualitative descriptive study was utilized to explore students’ experience attending the workshops and suggestions for improving the content to better meet the needs of nursing students. This was done through techniques that give voice and articulate participant perspectives in this study. Data collection was carried out through face-to-face semi-structured interviews with participants. The purpose of the interviews was to understand the participants’ perceptions of the impact of the workshops on their mental well-being.

### Participant recruitment and selection

Participants were recruited from the fourth and final semester of the Practical Nursing (PN) Program of a community college that enrolls approximately 190 students per year. This group was selected for two reasons. First, students would be shortly entering the nursing workforce and would benefit from learning strategies to support well-being and reduce burnout. Second, students in their final semester are exposed to significant academic and clinical demands that have been shown to increase stress and risk of burnout. Participants in the interview were recruited from the PN group who participated in the quantitative phase of the study [17]. For participation, there were no other restrictions placed on gender, ethnicity, or other demographic factors.

To introduce the study to students, a notice was posted on the school of nursing website, posters were displayed within nursing areas, and in-person recruitment was conducted during the “Nursing Care” course to inform students about the study. The principal investigator attended all course sections and provided an overview of the study, the time commitment, potential risks, and benefits to the students. Students had an opportunity to ask questions and were assured that participation was voluntary, was not tied to a course grade or their academic progress, and that they could discontinue participation at any time without penalty. Students were informed that the two mental fitness workshops were embedded into the skills rotations of their mandatory lab skills course. Recruitment occurred from March 2024 to May 2024. Information about the interview study was provided to all survey participants, and interested students were invited to contact the research team directly. All students who completed the survey were eligible to participate in an interview. Five students expressed interest and completed an interview.

### Interview process

The semi-structured interviews were conducted one to two weeks after the final workshop due to the recruitment and scheduling process. The semi-structured interviews were 30–60 minutes long and conducted by non-teaching research assistants not known to the students. The interview guide was co-developed by the research team based on the study objectives, the principles of MMMF-N program, and literature related to nursing well-being, resilience, and mental fitness. After reviewing relevant theory, the guide was pilot-tested with two non-participant students for clarity. Interviews were conducted online using Microsoft Teams, audio recorded, and transcribed verbatim.

The semi-structured interview guide was organized into three domains: (1) participants’ background and responsibilities, including employment, living arrangements, and caregiving responsibilities; (2) reasons for participating in the MMMF program and experiences of participation, including barriers to workshop attendance; and (3) post-program experiences and perceptions of the program. Example questions included: “How have the workshops strengthened your well-being, resilience, and mental fitness?”, “How do you see yourself incorporating what you have learned into your nursing career and into your life?”, and “What changes do you think should be made to this program to better serve nursing students?” The interviews were intended to provide context and depth to findings obtained through the participant feedback forms. Given the exploratory nature of this pilot study and the limited number of interview participants, data saturation was not formally assessed.

### Feedback forms

Participants also completed feedback forms at the end of each workshop that outlined their thoughts and opinions about the workshop content, length of the sessions, suggestions for improvement, and what they have learned. A total of 49 completed forms were included in the analysis. The feedback form contained a mix of closed-ended and open-ended questions. The closed-ended questions assessed the participants’ satisfaction with the MMMF-N workshop series. Satisfaction was measured using a five-point Likert-type scale ranging from 1 (“very dissatisfied”) to 5 (“very satisfied”). The open-ended questions asked participants to identify the most helpful features of the workshops, including the topics or activities they enjoyed. Participants were also asked about the workshops’ strengths and weaknesses, and how the workshops can be improved for future students. The feedback forms provided breadth by capturing input from a larger group of workshop participants. Open-ended responses were analyzed alongside the interviews. Responses to the open-ended questions on the feedback forms were analyzed using the thematic framework developed from the interview data. The feedback responses aligned with the identified themes and did not generate additional codes or themes.

### Reflexivity

The broader research team included a faculty member who was involved in the development and implementation of the MMMF-N workshops and assisted with participant recruitment. Given their familiarity with the intervention and investment in its success, this individual did not participate in data collection or analysis. This decision was made to reduce the potential influence of prior assumptions regarding the program on the interpretation of findings. The research team included nursing faculty members with an interest in student well-being and mental health promotion. Although the investigators were not involved in delivering the workshops, they were professionally invested in curriculum innovation, which could influence interpretation of findings. To mitigate potential power imbalances, data collection was conducted by independent research assistants not known to participants. Reflexivity was supported through regular team discussions during analysis, deliberate attention to divergent views, and the use of reflexive thematic analysis, which emphasizes transparency in analytic decision-making. The researchers reflected on how their professional backgrounds, experiences, and assumptions regarding student well-being and mental health could shape the interpretation of participants’ account.

### Rigor

NVivo 14 was used to support data organization and analysis [18]. Qualitative data obtained from interviews and open-ended feedback form responses were analyzed using reflexive thematic analysis, applying the six phases described by Braun and Clarke: [19] (1) familiarizing oneself with the data, (2) generating codes, (3) constructing themes, (4) reviewing potential themes, (5) defining and naming themes, and (6) producing the report.

Three research team members participated in coding and analysis: the principal investigator (LF) and two research assistants (JA and AD). LF is an early career researcher with training and experience in qualitative and mixed-methods research, including thematic analysis, content analysis, and interview-based methodologies. LF taught in the PN program but did not teach the cohort included in this study, had no evaluative relationship with the participants, and was not involved in conducting the interviews. Her role was limited to the analysis and interpretation of qualitative data. JA is a PhD student with training in qualitative and quantitative research methods. At the time of the study, JA had no affiliation with the PN program and no prior relationship with study participants. AD was a student research assistant enrolled in the Bachelor of Nursing program at the same institution. He had no teaching, supervisory, grading, or evaluative relationship with participants.

Several processes were followed to ensure rigor of analysis. A code book was maintained throughout the analytic process to support individual and collective understandings of code, categories, and eventually themes. Regular reflexive discussions were held among the research team to discuss coding decisions, fine code definitions, and develop themes. Any difference in coding or interpretation was resolved through discussion and consensus. Analytic decisions and theme revisions were documented throughout the study as an audit trail. Rich participant accounts were used to guide interpretation and ensure that themes remained grounded in the data. Additionally, the feedback form and interview transcripts were both analyzed during theme development. Although data saturation was not formally assessed, themes generated from the interviews were corroborated through analysis of the open-ended feedback form responses, which aligned with the thematic framework and did not generate additional codes or themes.

## Results

Our sample was comprised of 49 practical nursing students (see Table 2). There were 20 participants (41%) in the 18–24 age group with 22 participants (45%) being 25–34 years of age. The majority (92%) of participants were female and domestic (80%) students. Prior to entering the Practical Nursing program, participants had varying levels of educational attainment. Twenty-two (45%) participants reported a diploma as their highest level of education; 13 (27%) reported a high school diploma, while 11 (22%) reported a previous degree. Nursing was the first career for 57% of the participants.

**Table 2:** Participant demographics.

Demographic Characteristic	Frequency
	(n = 49)
**Age**	
18-24	20
25-34	22
35-44	4
45 and over	3
**Gender**	
Male	4
Female	45
**Type of Student**	
Domestic	39
International	10
**Education Prior to Admission to the Practical Nursing Program**	
High School	13
Diploma	22
Degree	11
Graduate	3
**Nursing as First Career**	
No	21
Yes	28

### Qualitative Findings

Feedback forms (n = 49) and interviews (n = 5) on the mental fitness workshops revealed four themes: 1) From ‘Not noticing’ to ‘Naming stressors’; 2) Reframing Self-care as Professional Competence; 3) Building Peer Connections and Mutual Support; and 4) Implementation Learnings.

Theme 1: From ‘Not noticing’ to ‘Naming stressors.’ Participants shared that attending the workshops had a positive impact on their understanding of mental health challenges currently affecting the nursing profession and nursing students: “Mental health during this nursing program, I just feel like that's a thing. That's a thing I have to emphasize now, but before that I didn't notice that” (Participant 2). Prior to the workshops, students had not fully considered the realities of the current nursing work environment or its impact on mental well-being. This realization encouraged participants to reflect on their well-being and learn strategies to improve their mental fitness. As one student shared, “After participating in this program. I think more about my mental issues, using more strategies to have a good mood, to have a positive attitude” (Participant 2). Students also experienced “lightbulb moments” during the workshops, where they began to make meaningful connections between their own experiences of stress and mental exhaustion and those of their clients. This shift in perspective enhanced their empathy and understanding and allowed them to recognize signs of stress in others while providing care: “So be kind to everyone. So yeah, this does help. This will help me in my career as well. Now I don't really judge people, right? I say okay, if he's angry at me, maybe he's just having a bad day” (Participant 1).

In addition to developing a greater awareness of burnout in the nursing profession, participants shared that the workshop activities helped them better assess their own mental fitness: “The very first workshop, that workshop made me do those surveys, to know where I am standing when it comes to mental fitness.” (Participant 5). Recognizing their current state of mental fitness played a crucial role in fostering “mental readiness,” which gave students the confidence to better navigate challenges and instilled hope for their growth in future nursing roles.

Theme 2: Reframing Self-care as Professional Competence*:* Increased awareness of mental fitness led participants to develop a deeper appreciation for the importance of self-care. Many participants mentioned improvements in their self-care routines after attending the workshops and expressed a strong intention to carry these practices forward into their future nursing careers: “I really understood the time management and time for self. Do something you like to help you to decompress, you know, your stress and anxiety level” (Participant 5). This insight also prompted participants to recognize the widespread lack of self-care within the nursing profession, as reflected in their observations of practicing nurses in clinical settings, As Participant 5 mentioned:

I'm seeing what is happening to my preceptors. The nurses working in the area where I am, being short of staff they have work overload, so I think it will help me to take it easy and do not take extra hours so that I don't find myself in those sorts of situations.

Some participants shared that the self-reflection encouraged by the workshops led to the realization that they were in a stronger place than they had believed before: “I always used to think I was just surviving, but after attending the workshop, I realized I’m not just surviving, I’m thriving” (Participant 1). Through the workshops, participants gained a greater awareness of their own resilience and the importance of caring for their mental health. Many participants shared that they had begun applying concepts from the workshops to their self-care practices: “After participating in this program, I’ve become more mindful of my mental health, using more strategies to improve my mood and maintain a positive attitude” (Participant 2). Maintaining a strong social support system was also viewed as an important component of mental fitness: “We also have to maintain good relationships with family and friends, take care of our physical fitness, and build confidence and resilience. Yeah, that’s a goal for nursing students” (Participant 2). These reflections underscore the value of fostering meaningful connections with peers and loved ones to support mental health and well-being.

Theme 3: Building Peer Connections and Mutual Support: Many participants reported experiencing more meaningful connections with their peers through participation in the workshops. As Participant 4 noted:

It’s important to realize that we’re not alone in this, and we’re not the only ones experiencing these struggles. That’s why it’s important for people to express themselves. At least when we share what we’re feeling, our stress starts to go down.

Small group discussions, created an opportunity for students to share experiences, listen to others, and engage in supportive conversations about the challenges of nursing education. Several students commented on how these small groups fostered deeper connections with peers and emphasized that this format made it easier to engage and share their experiences: “People feel more relaxed to face just three or two people......They feel more comfortable expressing themselves. That adds a lot of quality to the experience” (Participant 4). By the end of the workshops, participants expressed a sense of connection and mutual support, realizing they were not alone in their struggles: “We did a circle discussion, which is very important. You can share your experiences and also listen to others. You realize you are not alone, and you can gain a lot from others and express yourself too” (Participant 4). These interactive discussions and group activities allowed students to share their stories, learn from one another, and build rapport. The workshops provided tools not only for individual growth but also for recognizing and supporting the mental fitness of fellow students, reinforcing a sense of community and shared resilience.

Although participants did identify that the structure of the workshop facilitated connection among participants, most participants felt that there needed to be more small group activities. Students suggested that this type of activity was the most valuable to them, allowing connection with the content and their student colleagues: “Group discussion.... that's most important” (Participant 2). Having interactive discussions allowed participants to voice and acknowledge previously unrecognized exhaustion and hardship that they were facing in the nursing program. This let students feel engaged and supported, as they shared their lived experiences.

Theme 4: Implementation Learnings*:* Overall, participants found the content and activities to be highly beneficial in promoting self-awareness, and meaningful conversations about mental fitness and well-being. Students indicated that the workshops helped them connect with their “authentic self” through concepts such as the 6Fs and provided strategies for “taking care” of themselves during stressful times. As they engaged in discussions about stress, they gained a deeper understanding of both their own mental well-being and that of their peers. Students recommended incorporating more in-depth discussions about mental health, including the underlying causes and contributing factors. They expressed particular interest in exploring the “problems” that can lead to mental health challenges and the “symptoms” that may signal a decline in mental well-being: “What exactly is mental fitness, where I am when it comes to mental fitness, what I can do to make it better or what are those factors that are making it worse” (Participant 1).

Balancing multiple responsibilities including academic studies, work, clinical assignments, and familial obligations took a toll on student’s mental health, which emphasized the importance of developing coping strategies to prevent burnout: “I want to know more about mental health because when you are studying full time and you are a single parent, you're taking care of your child, you're taking care of all the house expenses and everything and your mental health, you cannot be mentally fit” (Participant 3). Participants indicated a strong interest in learning practical stress management techniques, such as engaging in hobbies, physical activity, and other coping strategies that can support them during high-stress periods.

Also, many students shared that they were able to attend the workshops primarily because they were integrated into their mandatory lab skills day: “To be very honest, it was because it was mandatory. That’s why people attended” (Participant 1). Students suggested that offering the workshops virtually could help increase accessibility for future sessions. This flexibility would allow students to join from various locations—whether commuting, at clinical, or at home: “They can just join the workshop even if it's mandatory—they can join from anywhere” (Participant 1). Participants also expressed that the workshops could be more beneficial for nursing students if they were offered at the start of the program in semester one. Given the demands of the program and the burnout that occurs, there was a shared consensus among some participants that the workshops should be introduced earlier in the PN program to offer tools and resources to reduce burnout. As Participant 5 mentioned:

They should have that workshop from semester one incorporated at some point in each semester, going down the line to semester four. Because I think students will need this kind of workshop from semester one to help them cope with mental health and stress duringstudy.

However, many participants indicated that the timing in the fourth semester was good given that they were completing their consolidation and entering the nursing workforce soon.

## Discussion

Results suggest that the MMMF-N workshops were well received and helpful for students during the final semester of their nursing program. Engagement in the workshops improved students’ ability to understand and identify burnout through topics like “The Profession of Nursing and Stress” and “Being aware of who I am”, allowing for greater awareness of their own mental health status. Reflective activities that were part of the workshops, such as the Six F’s of Health and Well-being framework, appeared to promote self-reflection and awareness of factors influencing their mental fitness and well-being. This finding aligns with literature suggesting that reflective and resilience-focused interventions can enhance self-awareness and psychological resilience among nursing students [20,21]. Also, short-term resilience building programs that focus on stress management, similar to the workshops in this study, can effectively equip students with strategies to recognize and manage stressors, reducing exhaustion and improving well-being during periods of intense academic stress [20–23].

What also emerged in this study was an improvement in participants’ recognition of distress in their clients. Following the workshops, some students reported an increased ability to recognize emotional distress in their clients and expressed enhanced empathy in clinical interactions. Evidence suggests that when you promote resilience at the individual level there is a correlation with improved client-centered care, aligning with relational and person-centered nursing models, which emphasize empathy as a fundamental professional attribute [20,23]. Additionally, the workshops fostered a sense of connection and community. Students expressed appreciation for the opportunity to engage in small-group discussions during the workshops and advocated for the inclusion of more peer-led, interactive elements. These insights are supported by previous research indicating that group-based and peer-supported interventions not only enhance engagement but also reduce feelings of isolation [20,24,25], which highlights the importance of social connection among nursing students [21,22,26]. Embedding brief, structured mental health promotion interventions within professional training programs may represent a feasible strategy for supporting psychological well-being and resilience. Such preventive approaches align with broader workforce mental health priorities and may contribute to sustainable coping practices in high-stress health professions.

Self-care was a introduced in the workshops through activities like “Identifying self-care strategies and developing my personal Mastering My Mental Fitness action / self-care plan.” However, participants mentioned that they wanted to learn more strategies to improve their mental fitness and reduce burnout. Complementary approaches, including yoga, mindfulness-based interventions, aromatherapy, and music therapy have shown promise in supporting psychological well-being and reducing stress among health professional students [27]. Existing evidence indicates that these types of targeted interventions play a vital role in supporting nursing students’ psychological well-being. Teaching some of these strategies would be beneficial to assist students in reducing their levels of burnout.

Having mental fitness workshops embedded throughout the nursing program, starting at semester one, was a recommendation of participants. Research does show that burnout increases as students’ progress through their nursing education [22], which aligns with providing these workshops earlier in the nursing curriculum to set students up for success in later semesters. However, despite participants being in their final semester, improvements observed in this study underscore that mental fitness interventions can remain impactful even at more advanced and demanding stages of training. While some scholars suggest that sustained long-term interventions are necessary to observe significant changes [25], our findings indicate that even two brief, yet structured sessions can effectively influence students’ attitudes, awareness, and coping strategies.

These findings support the early integration of mental fitness and well-being education within nursing curricula. Teaching self-care strategies early on may help establish a more sustainable professional foundation, equipping students to better navigate the evolving demands of healthcare [28]. Embedding this content longitudinally may not only support academic and emotional success but may help prepare students for the complex, relational aspects of nursing practice. Finally, offering workshops in hybrid or virtual formats could further increase accessibility for students by balancing coursework, clinical placements, employment, and family responsibilities. These flexible delivery models may help ensure broader participation and long-term impact across diverse student populations.

### Future research

Although students provided feedback on topics and activities they would want to see in future workshops, it would be good to conduct a needs assessment to inform tailored workshops that meet student needs. Next steps might also include a longitudinal study of mental fitness programming across the nursing program and compare the outcomes of in-person, virtual, and hybrid models to identify the most effective format.

### Limitations of the study

The findings from this study suggest that the workshops may have contributed to improvements in students’ mental fitness. However, the small interview sample size and a predominately female participant population does limit the generalizability of the results. Second, only a small subset of students agreed to participate in the semi-structured interviews, and it is possible that those students may have had a particular interest in mental well-being, which may have influenced their decision to engage in an interview. Finally, the evaluation period may not have been sufficient to capture changes. The intervention period was short, but the timing of the two workshops was chosen for this study due to the time commitments of students and the risk that students would not participate in a longer program. A longer follow-up period might reveal more substantial or sustained changes.

## Conclusion

Our research contributes to the Canadian literature by exploring interventions for improving mental fitness and reducing burnout among nursing students. As academic demands continue to increase in nursing programs, burnout will continue to be a concern among nursing students. Given this trend, there is a growing need to equip students with skills that enhance mental fitness. This study demonstrated that integrating two mental fitness workshops into the nursing curriculum appears to contribute to greater awareness of burnout and mental health challenges, greater self-reflection, and recognition of the importance of self-care and peer support. These findings highlight the potential of brief, curriculum‑embedded interventions to support mental health promotion and early burnout prevention among nursing students, contributing to more sustainable transitions into professional practice.
