# Supplementary material for: Implementing a mental fitness workshop in a Canadian Practical Nursing Program: A qualitative descriptive study
Source: PLOS Ment Health. 2026 Oct 28;3(10):e0000760. doi: 10.1371/journal.pmen.0000760 (PMC13716164; doi:10.1371/journal.pmen.0000760)
Supplement: Supplementary file 1 [file pmen.0000760.s002.docx]

Karli Montague-Cardoso
Staff Editor

PLOS Mental Health

September 11, 2026

Re: PMEN-D-26-00238

Dear Dr. Montague-Cardoso,

Thank you for the opportunity to revise our manuscript *Implementing a mental fitness workshop in a Canadian Practical Nursing Program: A qualitative descriptive study.*

Following this letter are the reviewer’s comments with our responses in italics, including how and where the text was modified. Changes were made in the manuscript, and the revised manuscript was uploaded. The revision has been developed in consultation with all coauthors, and each author has given approval to the final form of this revision.

Thank you for your consideration.

Sincerely,

Linda Frost PhD, NP

**Journal Requirements**

1. Please amend your detailed Financial Disclosure statement. This is published with the article. It must therefore be completed in full sentences and contain the exact wording you wish to be published.

i. Please clarify all sources of financial support for your study. List the grants, grant numbers, and organizations that funded your study, including funding received from your institution. Please note that suppliers of material support, including research materials, should be recognized in the Acknowledgements section rather than in the Financial Disclosure.
ii. State the initials, alongside each funding source, of each author to receive each grant. For example: "This work was supported by the National Institutes of Health (####### to AM; ###### to CJ) and the National Science Foundation (###### to AM)."
iii. State what role the funders took in the study. If the funders had no role in your study, please state: “The funders had no role in study design, data collection and analysis, decision to publish, or preparation of the manuscript.”
iv. If any authors received a salary from any of your funders, please state which authors and which funders.

*We have revised the Financial Disclosure statement to provide complete information regarding the funding source, recipient author initials, the role of the funder in the study, and salary support in accordance with journal requirements.*

2. Please send a completed 'Competing Interests' statement, including any COIs declared by your co-authors. If you have no competing interests to declare, please state "The authors have declared that no competing interests exist". Otherwise please declare all competing interests beginning with the statement "I have read the journal's policy and the authors of this manuscript have the following competing interests:"

*We confirm that all authors have reviewed the journal's competing interests policy and have no competing interests to declare. The Competing Interests statement has been included in the manuscript and reads: “The authors have declared that no competing interests exist.”*

3. In the online submission form, you indicated that “Due to the sensitive and personal nature of the qualitative data, interview transcripts and raw feedback cannot be shared publicly. De‑identified excerpts supporting the findings are included within the manuscript. Additional contextual information may be made available from the corresponding author upon reasonable request, subject to ethical approval and participant confidentiality.”.

All PLOS journals now require all data underlying the findings described in their manuscript to be freely available to other researchers, either
1. In a public repository,
2. Within the manuscript itself, or
3. Uploaded as supplementary information.

This policy applies to all data except where public deposition would breach compliance with the protocol approved by your research ethics board. If your data cannot be made publicly available for ethical or legal reasons (e.g., public availability would compromise patient privacy), please explain your reasons by return email and your exemption request will be escalated to the editor for approval. Your exemption request will be handled independently and will not hold up the peer review process but will need to be resolved should your manuscript be accepted for publication. One of the Editorial team will then be in touch if there are any issues.

*We have revised the Data Availability section and will send an email requesting an exemption.*

4. Please ensure that your Ethics Statement is available in its entirety at the beginning of your Methods section, under a subheading 'Ethics Statement'.

*We have revised the manuscript to include a dedicated Ethics Statement subsection at the beginning of the Methods section. The statement includes details regarding Research Ethics Board approval, informed consent procedures, voluntary participation, and measures taken to protect participant confidentiality.*

5. We do not publish any copyright or trademark symbols that usually accompany proprietary names, eg (R), (C), or TM (e.g. next to drug or reagent names). Please remove all instances of trademark/copyright symbols throughout the text, including Mental Fitness®.

*We have removed any copyright symbols in the manuscript.*

**Reviewer # 1**

1. **Section: Introduction and Background**
   The introduction provides a useful overview of the concept of mental fitness; however, the manuscript would benefit from a more explicit discussion of the broader framework of mental health promotion. Mental fitness appears to be the central intervention component, yet its relationship with mental health promotion is not sufficiently developed. Mental health promotion encompasses strategies aimed at strengthening personal, psychological, and social resources, whereas emotional well-being is generally considered an outcome associated with improved psychological adaptation and a lower risk of mental health difficulties. Clarifying these conceptual relationships would strengthen the theoretical foundation of the study.

*We agree and have added mental health promotion into the Introduction to more clearly situate mental fitness within the broader framework of mental health promotion. Specifically, we added text describing mental health promotion as an approach that strengthens personal, psychological, and social resources to support well-being and resilience. We also clarified that mental fitness*

2. Furthermore, in line 105, the authors state that the study is particularly relevant within the context of the current health crisis. It is unclear to which health crisis they are referring. Please specify whether this statement relates to: The post-pandemic context in Canada, challenges in healthcare workforce sustainability, increasing mental health concerns among students, or another specific context. Additional contextualization would enhance the rationale for the study.

*We agree that it is not clear. We clarified the study context by replacing the nonspecific reference to the “current healthcare crisis” with a description of the increasing mental health concerns among post-secondary students and the growing demands facing the nursing workforce in Canada.*

**Section: Study Design and Methodological Clarity**
The manuscript is presented as a qualitative descriptive study. While the design appears appropriate, several methodological details require further clarification.

3. Participant Selection
   In lines 143-144, one inclusion criterion states:
   “2) that they are likely experiencing burnout from program demands.”
   It is unclear how students meeting this criterion were identified.
   Please clarify:
   What indicators or procedures were used to determine that students were likely experiencing burnout?
   Was burnout formally assessed using a validated instrument?
   Was this determination based on self-identification, faculty observation, self-report, or another mechanism?

*We have clarified the rationale for recruiting fourth-semester Practical Nursing students. Specifically, we indicated that these students were nearing entry into the nursing workforce and were therefore an appropriate population for exploring mental fitness strategies. We also revised the wording to avoid assuming that participants were experiencing burnout and instead noted that final-semester nursing students are exposed to substantial academic and clinical demands that have been associated with increased stress and risk of burnout.*

4. Greater transparency regarding participant selection is needed.
   Reflexivity and Research Team
   Qualitative rigor would be enhanced through a more detailed reflexivity statement.
   Please clarify:
   How many researchers were involved in coding and analysis? What were their professional backgrounds and relationships to the participants?

How were disagreements resolved during the analytical process?

Were reflexive discussions or audit trails maintained?

*We have added information to the Rigor section that outlines the researchers experience in qualitative research and relationship to the participants. We have also added that we had regular reflexive discussions to discuss the coding and that disagreements were managed through consensus.*

**Description of Data Collection Procedures**
The manuscript reports the use of semi-structured interviews; however, insufficient information is provided regarding the interview guide.
It would be valuable to present:.

5. Whether the interview guide was piloted or revised during the study.

*This information was already mentioned in initial manuscript on lines 135-136. We have moved it now to be in the new section named “Interview Process” that we created in Design and Participation.*

6. The main domains explored during interviews. Examples of questions used in each domain Including a sample of interview questions, either within the manuscript or as supplementary material, would improve transparency and reproducibility.

*We have included more information on the interview guide with sample questions in the Design and Participant. This section is now divided into three parts to provide more detail on the interviews and feedback forms.*

**Results**
Participant Characteristics (Table 2)
Race
Table 2 includes participants’ race/ethnicity.
Please clarify:
Why was this variable collected?

7. How does it contribute to understanding participants’ experiences of the workshops?
   Was race considered analytically during interpretation of findings?
   If this variable was not relevant to the analysis, the rationale for its inclusion should be explained.

*We thank the reviewer for this observation. Race/ethnicity data was collected as part of a larger mixed-methods study examining factors associated with burnout among practical nursing students, where demographic variables were included in quantitative analyses. However, race/ethnicity was not used as an analytical variable in the qualitative component of the study, nor did it contribute substantively to the interpretation of participants' experiences of the workshops. To avoid potential confusion, we have removed race/ethnicity from Table 2 and revised the participant characteristics section accordingly.*

8. Educational Level. The meaning of the variable “Educational Level” is unclear. Given that all participants were recruited from the fourth and final semester of the Practical Nursing program, it is difficult to understand how educational level varied across participants.

*We thank the reviewer for this observation. Educational level referred to participants' highest level of education completed prior to entry into the Practical Nursing program. We have clarified this in the Results section and clarified this in Table 2*

9. Additional clarification is required.
   It may also be informative to report whether participants had previous healthcare or employment experience, as this factor could influence perceptions of stress, resilience, and mental health.

*We did not collect information about their previous career and employment experience. We only collected whether nursing was their first career. This will definitely be something we collect in future studies.*

10. Feedback Forms and Interview Sample
    The manuscript reports analysis of:
    Feedback forms (n = 49) and interviews (n = 5).
    However, several aspects are insufficiently described.
    Please clarify:

What specific questions were included in the feedback forms?

Were the forms open-ended, closed-ended, or mixed?

How were the responses incorporated into the qualitative analysis?

Why were only five students selected for interviews?

*We have added information on lines 169-172 to address this comment.*

11. How were these participants recruited?

*We have added information on lines 157-160 that indicates that participants were recruited from our larger quantitative study.*

12. What selection criteria were applied?

*We have added on lines 169-172 that all participants who completed the survey were eligible and invited to participate in an interview. However, only 5 students responded and agreed to participate.*

**Consistency Between Reported Themes and Findings**
The manuscript reports that the analysis revealed four themes:
1. Enhanced Awareness of Mental Health Challenges
2. Importance of Self-care
3. Building Supportive Connections
4. Perceptions Regarding the Workshops
However, the themes presented in the Results section are:
1. From “not noticing” to “naming stressors”
2. Reframing self-care as professional competence
3. Peer visibility reduces isolation
4. Implementation learnings

*We thank the reviewer for identifying this inconsistency. During manuscript revision, the themes were refined to more accurately reflect participants' experiences and the interpretive nature of the analysis. However, the summary statement preceding the Results section was not updated accordingly. We have revised the manuscript to ensure consistency throughout and now report the final themes as: (1) From "not noticing" to "naming stressors"; (2) Reframing self-care as professional competence; (3) Peer visibility reduces isolation; and (4) Implementation learnings.*

13. These two sets of themes appear conceptually related but are presented using different terminology.
    If the latter represents the final analytical framework emerging from data analysis, the authors should use this wording consistently throughout the manuscript. Alternatively, they should clearly explain the relationship between the broader conceptual categories and the final themes presented in the findings.
    Consistency across sections is essential for methodological coherence.

    **Reporting Quality**
    COREQ Reporting Standards
    Because this is a qualitative study, the manuscript would benefit from closer alignment with the Consolidated Criteria for Reporting Qualitative Research (COREQ).
    In particular, greater detail is needed regarding:
14. Research team characteristics and reflexivity.
    *We have added information in the Rigor section about our team characteristics and revised and added to the information under Reflexivity.*
15. Participant recruitment procedures.

*We have added information on lines 157-160 that indicates that participants were recruited from our larger quantitative study.*

16. Interview guide development.

*We have added information on how the interview guide was developed in the Interview Process section under Design and Participants.*

17. Data saturation.

*We have added information to the Interview Process (under Design and Participants) and Rigor sections clarifying that data saturation was not formally assessed and could not be assumed given the small number of participants who completed interviews. To enhance the trustworthiness of the findings, we analyzed open-ended responses from the participant feedback forms alongside the interview data. The feedback form responses aligned with the thematic framework developed from the interviews and did not generate additional codes or themes, providing triangulation across qualitative data sources.*

18. Coding procedures.

*We have added information in the Rigor section under Design and Participants*

19. Analytical decision-making processes.

*We have added information in the Rigor section under Design and Participants*

Explicit reference to COREQ adherence would strengthen the credibility and transparency of the study.

**Reviewer #2**

20. Authors do a more thorough analysis of existing literature as there are a number of publications specific to wellness/mental health interventions among Canadian nursing students (see for example Ciezar Andersen, S., Campbell, T., White, D., & King-Shier, K. (2024). An intervention to improve mental and physical health of undergraduate nursing students. Canadian Journal of Nursing Research, 56(3), 317-328. AND Ciezar-Andersen, S. D., Hayden, K. A., & King-Shier, K. M. (2021). A systematic review of yoga interventions for helping health professionals and students. Complementary Therapies in Medicine, 58, 102704.

*We have incorporated these two references in the discussion section of the manuscript.*

21. Specifically, authors only refer to data from the five interviews (need to specify when these took place in relation to the intervention), and do not draw on information gained from the feedback forms (n=49).

*To provide greater clarity, we have revised the manuscript to explain that responses to the open-ended feedback questions were analyzed using the thematic framework developed from the interview data. Feedback form responses aligned with the themes identified in the interviews and did not generate any additional codes or themes.*

22. Lines 70-74: no clear link is made between stress and burnout. Need more depth to explain what is meant by "stress students experience fluctuates over time" - evidence shows that nursing students report significant stress in all of their program years- need to do a more through background review of existing literature.

*We agree that the relationship between stress and burnout required further clarification. The background section has been revised to provide a more comprehensive discussion of the literature demonstrating that nursing students experience significant stress throughout their education, although the nature and intensity of stressors may change across program years. We have also strengthened the explanation of how persistent exposure to academic and clinical stressors can contribute to burnout and negatively affect students' mental well-being and readiness for professional practice.*

23. Line 85 "that help people feel better"- according to what criteria? self-report?
    *We agree that the original wording was overly simplistic and did not adequately reflect the concept of mental fitness. To address this concern, we revised the text to more clearly align with our cited definition of mental fitness*
24. Line 99-100: again, need more through literature review of existing evidence. See Andersen, S., Campbell, T., White, D., & King-Shier, K. (2024). An intervention to improve mental and physical health of undergraduate nursing students. Canadian Journal of Nursing Research, 56(3), 317-328.
25. Line 150- incomplete sentence

*The sentence has been revised for completeness and clarity by removing the word “including.”*

26. Line 231- first and only mention of PHYSICAL health

*We have removed “physical” from the sentence as this was not a significant factor mentioned by participants.*

27. Line 240- Peer CONNECTION not visibility. There is no evidence to suggest feelings of isolation were reduced.

*We agree and have revised the theme to better reflect the participant’s experience.* *The revised theme emphasizes peer connection and mutual support rather than a reduction in isolation.*

28. Line 267- refer to stress management but this was not measured or inquired about.

*We agree and have removed the mention of “stress management.”*

29. Line 312- the reference is wrong, it has nothing to do with students or burnout.

*We agree that Braun and Clarke (2019) was not the most appropriate reference to support this statement, as it focuses on reflexive thematic analysis rather than the outcomes of reflective interventions. We have removed this citation and replaced it with literature examining reflective and resilience-focused interventions among nursing students. In addition, the text has been revised to more closely align with the findings of our study.*

30. Line 337- systematic reviews more specific are available- see Ciezar-Andersen, S. D., Hayden, K. A., & King-Shier, K. M. (2021). A systematic review of yoga interventions for helping health professionals and students.

*We agree that more specific evidence was warranted. We revised the statement to cite systematic review evidence related to complementary interventions among health professional students and practitioners, including the review by Ciezar-Andersen et al. (2021), rather than relying on broader post-secondary student literature.*

31. Line 348-350- not clear when the data was collected in relation to the intervention timeline. Immediately after or weeks later?

*We have identified this now in the Participant and Design section in lines 174-175.*

32. Line 383- claim of "enhanced coping strategies" is not supported by the findings.457-460-

*We thank the reviewer for this observation and agree that the findings do not directly*	*demonstrate enhanced coping strategies. We have revised the Discussion to more*	*accurately reflect the findings by focusing on participants' increased awareness of*	*burnout and mental health challenges, greater self-reflection, and recognition of the*	*importance of self-care and peer support, rather than improvements in coping strategies.*	*This revision ensures that our conclusions remain aligned with the qualitative findings.*

33. Review reference details and add DOI.

*We have reviewed the references and ensured that if a doi exists for that reference that it was added. There is no doi for the Rogers & Baker reference.*
