# Supplementary material for: Implementing a mental fitness workshop in a Canadian Practical Nursing Program: A qualitative descriptive study
Source: PLOS Ment Health. 2026 Oct 28;3(10):e0000760. doi: 10.1371/journal.pmen.0000760 (PMC13716164; doi:10.1371/journal.pmen.0000760)
Supplement: Supplementary file 2 [file pmen.0000760.s003.docx]

Karli Montague-Cardoso
Staff Editor

PLOS Mental Health

October 1, 2026

Re: PMEN-D-26-00238

Dear Dr. Montague-Cardoso,

Thank you for the opportunity to revise our manuscript *Implementing a mental fitness workshop in a Canadian Practical Nursing Program: A qualitative descriptive study.*

Following this letter are the reviewer’s comments with our responses in italics, including how and where the text was modified. Changes were made in the manuscript, and the revised manuscript was uploaded. The revision has been developed in consultation with all coauthors, and each author has given approval to the final form of this revision.

Thank you for your consideration.

Sincerely,

Linda Frost PhD, NP

**Reviewer # 1**

1. The expression “current healthcare crisis” is still present in the manuscript, although the response letter indicates that this wording was revised. I recommend replacing this phrase with a more specific description of the context to which the authors are referring.
   *We agree and at the end of the introduction, before the Methods section, we have removed “current healthcare crisis” and replaced it with “current mental health challenges experienced by nursing students.”*
2. Please ensure complete consistency in the naming of Theme 3 throughout the manuscript. Different versions of the theme title appear in the revised documents and should be standardised.
   *We have revised the themes to be standardized in the document.*
3. A final proof-reading and editorial review would be beneficial, as a number of minor formatting and editing issues remain in the revised version.

*We have read through the manuscript and made formatting and editing changes throughout.*
