# Supplementary material for: Implementing a mental fitness workshop in a Canadian Practical Nursing Program: A qualitative descriptive study
Source: PLOS Ment Health. 2026 Oct 28;3(10):e0000760. doi: 10.1371/journal.pmen.0000760 (PMC13716164; doi:10.1371/journal.pmen.0000760)
Supplement: Peer review history

# Peer review: Decision Letter 0

**DOI:** 10.1371/journal.pmen.0000760.r001

**Authors:** Karli Montague-Cardoso

11 Aug 2026

PMEN-D-26-00238

Implementing a mental fitness workshop in a Canadian Practical Nursing Program: A qualitative descriptive study Integrating mental fitness into a Canadian Practical Nursing Program

PLOS Mental Health

Dear Dr. Frost,

Thank you for submitting your manuscript to PLOS Mental Health. I am sorry for the delay. After careful consideration of the reviewer reports, we feel that it has merit but does not fully meet PLOS Mental Health’s publication criteria as it currently stands. Therefore, we invite you to submit a revised version of the manuscript that addresses the points raised during the review process. You can find all comments at the end of this email.

Please submit your revised manuscript by Oct 09 2026 11:59PM. If you will need more time than this to complete your revisions, please reply to this message or contact the journal office at mentalhealth@plos.org. When you're ready to submit your revision, log on to https://www.editorialmanager.com/pmen/ and select the 'Submissions Needing Revision' folder to locate your manuscript file.

Please include the following items when submitting your revised manuscript:

- A letter that responds to each point raised by the editor and reviewer(s). You should upload this letter as a separate file labeled 'Response to Reviewers'.
- A marked-up copy of your manuscript that highlights changes made to the original version. You should upload this as a separate file labeled 'Revised Manuscript with Track Changes'.
- An unmarked version of your revised paper without tracked changes. You should upload this as a separate file labeled 'Manuscript'.

Guidelines for resubmitting your figure files are available below the reviewer comments at the end of this letter.

As the corresponding author, your ORCID iD is verified in the submission system and will appear in the published article. PLOS supports the use of ORCID, and we encourage all coauthors to register for an ORCID iD and use it as well. Please encourage your coauthors to verify their ORCID iD within the submission system before final acceptance, as unverified ORCID iDs will not appear in the published article. *Only*  the individual author can complete the verification step; PLOS staff *cannot*  verify ORCID iDs on behalf of authors.

We look forward to receiving your revised manuscript.

Kind regards,

Karli Montague-Cardoso

Staff Editor

PLOS Mental Health

**Journal Requirements:**

1. Please amend your detailed Financial Disclosure statement. This is published with the article. It must therefore be completed in full sentences and contain the exact wording you wish to be published.

i. Please clarify all sources of financial support for your study. List the grants, grant numbers, and organizations that funded your study, including funding received from your institution. Please note that suppliers of material support, including research materials, should be recognized in the Acknowledgements section rather than in the Financial Disclosure.

ii. State the initials, alongside each funding source, of each author to receive each grant. For example: "This work was supported by the National Institutes of Health (####### to AM; ###### to CJ) and the National Science Foundation (###### to AM)."

iii. State what role the funders took in the study. If the funders had no role in your study, please state: “The funders had no role in study design, data collection and analysis, decision to publish, or preparation of the manuscript.”

iv. If any authors received a salary from any of your funders, please state which authors and which funders.

2. Please send a completed 'Competing Interests' statement, including any COIs declared by your co-authors. If you have no competing interests to declare, please state "The authors have declared that no competing interests exist". Otherwise please declare all competing interests beginning with the statement "I have read the journal's policy and the authors of this manuscript have the following competing interests:"

3. In the online submission form, you indicated that “Due to the sensitive and personal nature of the qualitative data, interview transcripts and raw feedback cannot be shared publicly. De‑identified excerpts supporting the findings are included within the manuscript. Additional contextual information may be made available from the corresponding author upon reasonable request, subject to ethical approval and participant confidentiality.”.

All PLOS journals now require all data underlying the findings described in their manuscript to be freely available to other researchers, either

1. In a public repository,

2. Within the manuscript itself, or

3. Uploaded as supplementary information.

This policy applies to all data except where public deposition would breach compliance with the protocol approved by your research ethics board. If your data cannot be made publicly available for ethical or legal reasons (e.g., public availability would compromise patient privacy), please explain your reasons by return email and your exemption request will be escalated to the editor for approval. Your exemption request will be handled independently and will not hold up the peer review process, but will need to be resolved should your manuscript be accepted for publication. One of the Editorial team will then be in touch if there are any issues.

4. Please ensure that your Ethics Statement is available in its entirety at the beginning of your Methods section, under a subheading 'Ethics Statement'.

5. We do not publish any copyright or trademark symbols that usually accompany proprietary names, eg (R), (C), or TM  (e.g. next to drug or reagent names). Please remove all instances of trademark/copyright symbols throughout the text, including Mental Fitness®.

If the reviewer comments include a recommendation to cite specific previously published works, please review and evaluate these publications to determine whether they are relevant and should be cited. There is no requirement to cite these works unless the editor has indicated otherwise.

**Additional Editor Comments (if provided):**

[Note: HTML markup is below. Please do not edit.]

Reviewers' comments:

Reviewer's Responses to Questions

**Comments to the Author**

1. Does this manuscript meet PLOS Mental Health’s publication criteria?

Reviewer #1: Yes

Reviewer #2: Partly

**********

2. Has the statistical analysis been performed appropriately and rigorously?

Reviewer #1: Yes

Reviewer #2: N/A

**********

3. Have the authors made all data underlying the findings in their manuscript fully available (please refer to the Data Availability Statement at the start of the manuscript PDF file)?

The PLOS Data policy

Reviewer #1: No

Reviewer #2: No

**********

4. Is the manuscript presented in an intelligible fashion and written in standard English?

Reviewer #1: Yes

Reviewer #2: Yes

**********

**Reviewer #1:**  Reviewer Comments to the Authors

Thank you for the opportunity to review this manuscript. The topic is relevant and timely, particularly given the growing international interest in mental health promotion, psychological well-being, and resilience among nursing students. The manuscript addresses an important educational initiative and offers potentially valuable insights into the integration of mental fitness concepts within nursing education. However, several areas require clarification and further development to strengthen the methodological transparency and overall rigor of the study.

Major Comments

1. Introduction and Background

The introduction provides a useful overview of the concept of mental fitness; however, the manuscript would benefit from a more explicit discussion of the broader framework of mental health promotion.

Mental fitness appears to be the central intervention component, yet its relationship with mental health promotion is not sufficiently developed. Mental health promotion encompasses strategies aimed at strengthening personal, psychological, and social resources, whereas emotional well-being is generally considered an outcome associated with improved psychological adaptation and a lower risk of mental health difficulties. Clarifying these conceptual relationships would strengthen the theoretical foundation of the study.

Furthermore, in line 105, the authors state that the study is particularly relevant within the context of the current health crisis. It is unclear to which health crisis they are referring. Please specify whether this statement relates to:

The post-pandemic context in Canada, challenges in healthcare workforce sustainability, increasing mental health concerns among students, or another specific context.

Additional contextualization would enhance the rationale for the study.

2. Study Design and Methodological Clarity

The manuscript is presented as a qualitative descriptive study. While the design appears appropriate, several methodological details require further clarification.

Participant Selection

In lines 143-144, one inclusion criterion states:

“2) that they are likely experiencing burnout from program demands.”

It is unclear how students meeting this criterion were identified.

Please clarify:

What indicators or procedures were used to determine that students were likely experiencing burnout?

Was burnout formally assessed using a validated instrument?

Was this determination based on self-identification, faculty observation, self-report, or another mechanism?

Greater transparency regarding participant selection is needed.

Reflexivity and Research Team

Qualitative rigor would be enhanced through a more detailed reflexivity statement.

Please clarify:

How many researchers were involved in coding and analysis?

What were their professional backgrounds and relationships to the participants?

How were disagreements resolved during the analytical process?

Were reflexive discussions or audit trails maintained?

3. Description of Data Collection Procedures

The manuscript reports the use of semi-structured interviews; however, insufficient information is provided regarding the interview guide.

It would be valuable to present:

The main domains explored during interviews.

Examples of questions used in each domain.

Whether the interview guide was piloted or revised during the study.

Including a sample of interview questions, either within the manuscript or as supplementary material, would improve transparency and reproducibility.

Results

4. Participant Characteristics (Table 2)

Race

Table 2 includes participants’ race/ethnicity.

Please clarify:

Why was this variable collected?

How does it contribute to understanding participants’ experiences of the workshops?

Was race considered analytically during interpretation of findings?

If this variable was not relevant to the analysis, the rationale for its inclusion should be explained.

Educational Level

The meaning of the variable “Educational Level” is unclear.

Given that all participants were recruited from the fourth and final semester of the Practical Nursing program, it is difficult to understand how educational level varied across participants.

Additional clarification is required.

It may also be informative to report whether participants had previous healthcare or employment experience, as this factor could influence perceptions of stress, resilience, and mental health.

5. Feedback Forms and Interview Sample

The manuscript reports analysis of:

Feedback forms (n = 49) and interviews (n = 5).

However, several aspects are insufficiently described.

Please clarify:

What specific questions were included in the feedback forms?

Were the forms open-ended, closed-ended, or mixed?

How were the responses incorporated into the qualitative analysis?

Why were only five students selected for interviews?

How were these participants recruited?

What selection criteria were applied?

A more detailed description of these procedures would improve methodological transparency.

6. Consistency Between Reported Themes and Findings

The manuscript reports that the analysis revealed four themes:

1. Enhanced Awareness of Mental Health Challenges

2. Importance of Self-care

3. Building Supportive Connections

4. Perceptions Regarding the Workshops

However, the themes presented in the Results section are:

1. From “not noticing” to “naming stressors”

2. Reframing self-care as professional competence

3. Peer visibility reduces isolation

4. Implementation learnings

These two sets of themes appear conceptually related but are presented using different terminology.

If the latter represents the final analytical framework emerging from data analysis, the authors should use this wording consistently throughout the manuscript. Alternatively, they should clearly explain the relationship between the broader conceptual categories and the final themes presented in the findings.

Consistency across sections is essential for methodological coherence.

Reporting Quality

7. COREQ Reporting Standards

Because this is a qualitative study, the manuscript would benefit from closer alignment with the Consolidated Criteria for Reporting Qualitative Research (COREQ).

In particular, greater detail is needed regarding:

Research team characteristics and reflexivity.

Participant recruitment procedures.

Interview guide development.

Data saturation.

Coding procedures.

Analytical decision-making processes.

Explicit reference to COREQ adherence would strengthen the credibility and transparency of the study.

Overall Assessment

This manuscript addresses an important and highly relevant topic in nursing education and mental health promotion. The intervention appears promising and potentially transferable to other educational settings. Nevertheless, several methodological and reporting aspects require clarification before the findings can be fully appraised. Addressing the points raised above would significantly improve the transparency, rigor, and interpretability of the study.

Recommendation to the Editor

Major Revision

Confidential Comments to the Editor

This manuscript addresses an important topic related to mental health promotion and well-being among nursing students and presents a potentially valuable educational intervention. The topic is well aligned with the scope of PLOS Mental Health.

However, several methodological and reporting issues require substantial clarification before the study can be adequately evaluated. The manuscript would benefit from a stronger theoretical justification of the relationship between mental fitness, mental health promotion and emotional well-being. In addition, there are important gaps regarding participant selection, identification of students experiencing burnout, reflexivity, interview procedures, feedback forms, sampling decisions, and consistency in the presentation of qualitative themes.

Reporting of the qualitative methodology would be strengthened through closer adherence to the COREQ guidelines. While I did not identify major concerns regarding the relevance of the research question, the current version lacks sufficient methodological detail to ensure transparency and trustworthiness of the findings.

Therefore, I recommend Major Revision, allowing the authors the opportunity to address these issues and improve the clarity and rigor of the manuscript.

**Reviewer #2:**  Authors do a more thorough analysis of existing literature as there are a number of publications specific to wellness/mental health interventions among Canadian nursing students (see for example Ciezar Andersen, S., Campbell, T., White, D., & King-Shier, K. (2024). An intervention to improve mental and physical health of undergraduate nursing students. Canadian Journal of Nursing Research, 56(3), 317-328. AND Ciezar-Andersen, S. D., Hayden, K. A., & King-Shier, K. M. (2021). A systematic review of yoga interventions for helping health professionals and students. Complementary Therapies in Medicine, 58, 102704.

Specifically, authors only refer to data from the five interviews (need to specify when these took place in relation to the intervention), and do not draw on information gained from the feedback forms (n=49).

Lines 70-74: no clear link is made between stress and burnout. Need more depth to explain what is meant by "stress students experience fluctuates over time" - evidence shows that nursing students report significant stress in all of their program years- need to do a more through background review of existing literature.

Line 85 "that help people feel better"- according to what criteria? self-report?

Line 99-100: again, need more through literature review of existing evidence. See Andersen, S., Campbell, T., White, D., & King-Shier, K. (2024). An intervention to improve mental and physical health of undergraduate nursing students. Canadian Journal of Nursing Research, 56(3), 317-328.

Line 150- incomplete sentence

Line 231- first and only mention of PHYSICAL health

Line 240- Peer CONNECTION not visibility. There is no evidence to suggest feelings of isolation were reduced.

Line 267- refer to stress management but this was not measured or inquired about.

Line 312- the reference is wrong, it has nothing to do with students or burnout.

Line 337- systematic reviews more specific are available- see Ciezar-Andersen, S. D., Hayden, K. A., & King-Shier, K. M. (2021). A systematic review of yoga interventions for helping health professionals and students.

Line 348-350- not clear when the data was collected in relation to the intervention timeline. Immediately after or weeks later?

Line 383- claim of "enhanced coping strategies" is not supported by the findings.457-460- Review reference details and add DOI.

**********

what does this mean?). If published, this will include your full peer review and any attached files.

**Do you want your identity to be public for this peer review?**  If you choose “no”, your identity will remain anonymous but your review may still be made public.

For information about this choice, including consent withdrawal, please see our Privacy Policy

Reviewer #1: **Yes:**  Clara Isabel Posada Abadía

Reviewer #2: No

**********

[NOTE: If reviewer comments were submitted as an attachment file, they will be attached to this email and accessible via the submission site. Please log into your account, locate the manuscript record, and check for the action link "View Attachments". If this link does not appear, there are no attachment files.]

**Figure Resubmissions:**

# Author comment: Author response to Decision Letter 1

**DOI:** 10.1371/journal.pmen.0000760.r002

17 Sep 2026

Attachment Submitted filename: PLOS Mental Health Response to Reviewers.docx

# Peer review: Decision Letter 1

**DOI:** 10.1371/journal.pmen.0000760.r003

**Authors:** Karli Montague-Cardoso

29 Sep 2026

PMEN-D-26-00238R1

Implementing a mental fitness workshop in a Canadian Practical Nursing Program: A qualitative descriptive study Integrating mental fitness into a Canadian Practical Nursing Program

PLOS Mental Health

Dear Dr. Frost,

Thank you for submitting your revised manuscript to PLOS Mental Health. The editorial team and the reviewers found the manuscript to be significantly improved. We would like to request some final, minor revisions prior to accepting the manuscript. You can find the remaining comments below.

Please submit your revised manuscript by Oct 28 2026 11:59PM. If you will need more time than this to complete your revisions, please reply to this message or contact the journal office at mentalhealth@plos.org. When you're ready to submit your revision, log on to https://www.editorialmanager.com/pmen/ and select the 'Submissions Needing Revision' folder to locate your manuscript file.

Please include the following items when submitting your revised manuscript:

- A letter that responds to each point raised by the editor and reviewer(s). You should upload this letter as a separate file labeled 'Response to Reviewers'.
- A marked-up copy of your manuscript that highlights changes made to the original version. You should upload this as a separate file labeled 'Revised Manuscript with Track Changes'.
- An unmarked version of your revised paper without tracked changes. You should upload this as a separate file labeled 'Manuscript'.

Guidelines for resubmitting your figure files are available below the reviewer comments at the end of this letter.

As the corresponding author, your ORCID iD is verified in the submission system and will appear in the published article. PLOS supports the use of ORCID, and we encourage all coauthors to register for an ORCID iD and use it as well. Please encourage your coauthors to verify their ORCID iD within the submission system before final acceptance, as unverified ORCID iDs will not appear in the published article. *Only*  the individual author can complete the verification step; PLOS staff *cannot*  verify ORCID iDs on behalf of authors.

We look forward to receiving your revised manuscript.

Kind regards,

Karli Montague-Cardoso

Staff Editor

PLOS Mental Health

**Journal Requirements:**

If the reviewer comments include a recommendation to cite specific previously published works, please review and evaluate these publications to determine whether they are relevant and should be cited. There is no requirement to cite these works unless the editor has indicated otherwise.

Please review your reference list to ensure that it is complete and correct. If you have cited papers that have been retracted, please include the rationale for doing so in the manuscript text, or remove these references and replace them with relevant current references. Any changes to the reference list should be mentioned in the rebuttal letter that accompanies your revised manuscript. If you need to cite a retracted article, indicate the article’s retracted status in the References list and also include a citation and full reference for the retraction notice.

[Note: HTML markup is below. Please do not edit.]

Reviewers' comments:

Reviewer's Responses to Questions

**Comments to the Author**

Reviewer #1: (No Response)

**********

publication criteria?

Reviewer #1: Yes

**********

3. Has the statistical analysis been performed appropriately and rigorously?

Reviewer #1: Yes

**********

4. Have the authors made all data underlying the findings in their manuscript fully available (please refer to the Data Availability Statement at the start of the manuscript PDF file)?

The PLOS Data policy

Reviewer #1: Yes

**********

5. Is the manuscript presented in an intelligible fashion and written in standard English?

Reviewer #1: Yes

**********

**Reviewer #1:**  Thank you for the revised manuscript and the detailed response to the reviewers’ comments. The authors have addressed the majority of the concerns raised during the previous review and have substantially improved the manuscript. In particular, the additional information regarding the conceptual link between mental fitness and mental health promotion, participant recruitment, interview procedures, reflexivity, coding processes and the integration of feedback-form data have strengthened the methodological transparency and overall rigour of the study.

I have only a few minor comments remaining:

1. The expression “current healthcare crisis” is still present in the manuscript, although the response letter indicates that this wording was revised. I recommend replacing this phrase with a more specific description of the context to which the authors are referring.

2. Please ensure complete consistency in the naming of Theme 3 throughout the manuscript. Different versions of the theme title appear in the revised documents and should be standardised.

3. A final proof-reading and editorial review would be beneficial, as a number of minor formatting and editing issues remain in the revised version.

I believe the manuscript has improved considerably and is close to being suitable for publication, subject to these minor revisions.

**********

what does this mean?). If published, this will include your full peer review and any attached files.

**Do you want your identity to be public for this peer review?**  If you choose “no”, your identity will remain anonymous but your review may still be made public.

For information about this choice, including consent withdrawal, please see our Privacy Policy

Reviewer #1: **Yes:**  Clara Isabel Posada Abadía

**********

[NOTE: If reviewer comments were submitted as an attachment file, they will be attached to this email and accessible via the submission site. Please log into your account, locate the manuscript record, and check for the action link "View Attachments". If this link does not appear, there are no attachment files.]

**Figure Resubmissions:**

# Author comment: Author response to Decision Letter 2

**DOI:** 10.1371/journal.pmen.0000760.r004

2 Oct 2026

Attachment Submitted filename: PLOS Mental Health Response to Reviewers 2.docx

# Editor report: Decision Letter 2

**DOI:** 10.1371/journal.pmen.0000760.r005

**Authors:** Karli Montague-Cardoso

6 Oct 2026

Implementing a mental fitness workshop in a Canadian Practical Nursing Program: A qualitative descriptive study Integrating mental fitness into a Canadian Practical Nursing Program

PMEN-D-26-00238R2

Dear Dr. Frost,

We are pleased to inform you that your manuscript 'Implementing a mental fitness workshop in a Canadian Practical Nursing Program: A qualitative descriptive study Integrating mental fitness into a Canadian Practical Nursing Program' has been provisionally accepted for publication in PLOS Mental Health.

Before your manuscript can be formally accepted you will need to complete some formatting changes, which you will receive in a follow up email. A member of our team will be in touch with a set of requests.

Please note that your manuscript will not be scheduled for publication until you have made the required changes, so a swift response is appreciated.

IMPORTANT: The editorial review process is now complete. PLOS will only permit corrections to spelling, formatting or significant scientific errors from this point onwards. Requests for major changes, or any which affect the scientific understanding of your work, will cause delays to the publication date of your manuscript.

If your institution or institutions have a press office, please notify them about your upcoming paper to help maximize its impact. If they'll be preparing press materials, please inform our press team as soon as possible -- no later than 48 hours after receiving the formal acceptance. Your manuscript will remain under strict press embargo until 2 pm Eastern Time on the date of publication. For more information, please contact mentalhealth@plos.org.

Thank you again for supporting Open Access publishing; we are looking forward to publishing your work in PLOS Mental Health.

Best regards,

Karli Montague-Cardoso

Staff Editor

PLOS Mental Health

***********************************************************

Reviewer Comments (if any, and for reference):
